# Zebrafish (Danio rerio) larvae as a predictive model to study gentamicin-induced structural alterations of the kidney

**DOI:** 10.1371/journal.pone.0284562

**Published:** 2023-04-20

**Authors:** Jan Stephan Bolten, Christine Tanner, Griffin Rodgers, Georg Schulz, Soledad Levano, Timm Weitkamp, Samuel Waldner, Ramya Deepthi Puligilla, Daniel Bodmer, Bert Müller, Jörg Huwyler

**Affiliations:** 1 Division of Pharmaceutical Technology, Department of Pharmaceutical Sciences, University of Basel, Basel, Switzerland; 2 Biomaterial Science Center, University of Basel, Basel, Switzerland; 3 Department of Biomedicine, University Hospital Basel, Basel, Switzerland; 4 Synchrotron SOLEIL, Saint-Aubin, France; Semmelweis University, HUNGARY

## Abstract

Nephrotoxicity is an important drug safety aspect to be assessed during drug discovery and development. To study renal toxicity, *in vitro* cell-based assays are often used. Unfortunately, translating the results of such cell assays to vertebrates including human remains challenging. Therefore, we aim to evaluate whether zebrafish larvae (ZFL) could serve as a vertebrate screening model to detect gentamicin-induced changes of kidney glomeruli and proximal tubules. To validate the model, we compared the results of ZFL with those obtained from kidney biopsies of gentamicin-treated mice. We used transgenic zebrafish lines expressing enhanced green fluorescent proteins in the glomerulus to visualize glomerular damage. Synchrotron radiation-based computed tomography (SRμCT) is a label-free approach providing three-dimensional representations of renal structures with micrometre resolution. Clinically used gentamicin concentrations induce nephrotoxicity and affect glomerular and proximal tubular morphology. Findings were confirmed in mice and ZFL. There was a strong correlation between fluorescent signals in ZFL, SRμCT- derived descriptors of glomerular and proximal tubular morphology and the histological analysis of mouse kidney biopsies. A combination of SRμCT and confocal microscopy provides unprecedented insights into anatomical structures of the zebrafish kidney. Based on our findings, we suggest to use ZFL as a predictive vertebrate screening model to study drug-induced nephrotoxicity and to bridge the gap between cell culture-based test systems and experiments in mammals.

## Introduction

The kidney is an essential excreting organ. Renal function comprises glomerular filtration, tubular excretion, and tubular reabsorption. Thereby, certain xenobiotics accumulate within the kidney and may cause nephrotoxicity at the vascular, glomerular, or tubular level. Examples of such nephrotoxic compounds include aminoglycosides, heavy metals, and antiviral agents [1]. In clinical settings, therapies with potentially nephrotoxic drugs need to be monitored, and doses have to be adjusted in the case of renal impairment. For instance, nephrotoxic side effects are common in patients treated with the antibiotic drug gentamicin. In 50% of patients, signs of nephrotoxicity can be detected after 14 days of drug therapy [2, 3]. Gentamicin-induced nephrotoxicity is thus clinically relevant and was described as a class effect of aminoglycosides leading to acute kidney injury [4]. The toxic effect is associated with receptor-mediated accumulation in renal epithelial cells by megalin/ cubulin [5, 6].

The problem of potential nephrotoxic side effects of drugs should be addressed during the early phases of preclinical development. Usually, *in vitro* cell-based assays are standard methods for safety and toxicity assessments [7]. They are cost-effective and provide mechanistic insights. However, *in vitro* assays do not cover certain renal functions, such as glomerular filtration [7, 8]. Therefore, findings must be confirmed in living organisms, which is a demanding task. Here, various challenges have to be addressed. Animal experiments are time-consuming, involve substantial costs and rely on *in vivo* experimentation with higher and more complex vertebrates, such as rats and mice. There is an urgent and unmet need to reduce, replace, and refine animal models (as described in the 3R principles) and establish animal-reducing (screening) *in vivo* test systems.

Previously we demonstrated that zebrafish larvae (ZFL) between two and four days post-fertilization (dpf) can be used as a predictive vertebrate *in vivo* screening model for nanomedicine [9]. ZFL are transparent, have a short generation time, and have the regulatory status of cell culture models up to 120 hours’ post-fertilization (hpf). They are widely used in developmental biology, toxicology, and pharmacology [10, 11] including nephrotoxicology [12, 13]. Intravenous *(i*.*v*.*)* administration of fluorescent substances allows for studying the pharmacokinetics and tissue distribution of test compounds [8, 14]. Semiquantitative assessment of systemic circulation and extravasation of intravenously injected drug formulations [14, 15] allows for extrapolation to higher vertebrates including rodents.

Previously, we could demonstrate that 3-to-4-day-old ZFL has fully developed renal functions [8]. The teleost pronephron shares high similarities with the function and anatomy of the vertebrate kidney [16, 17]. Indeed, ZFL were used to study aspects of nephrotoxicity on cellular and glomerular levels [12, 18, 19].

Nevertheless, it is still unclear how nephrotoxins affect excreting organs of the ZFL, especially with respect to the morphology of the pronephron. Therefore, the present study aimed to clarify whether ZFL can be used as a model for the morphological characterization of kidneys exposed to toxic drugs and for the extrapolation of such results to higher vertebrates.

**Fig 1:**
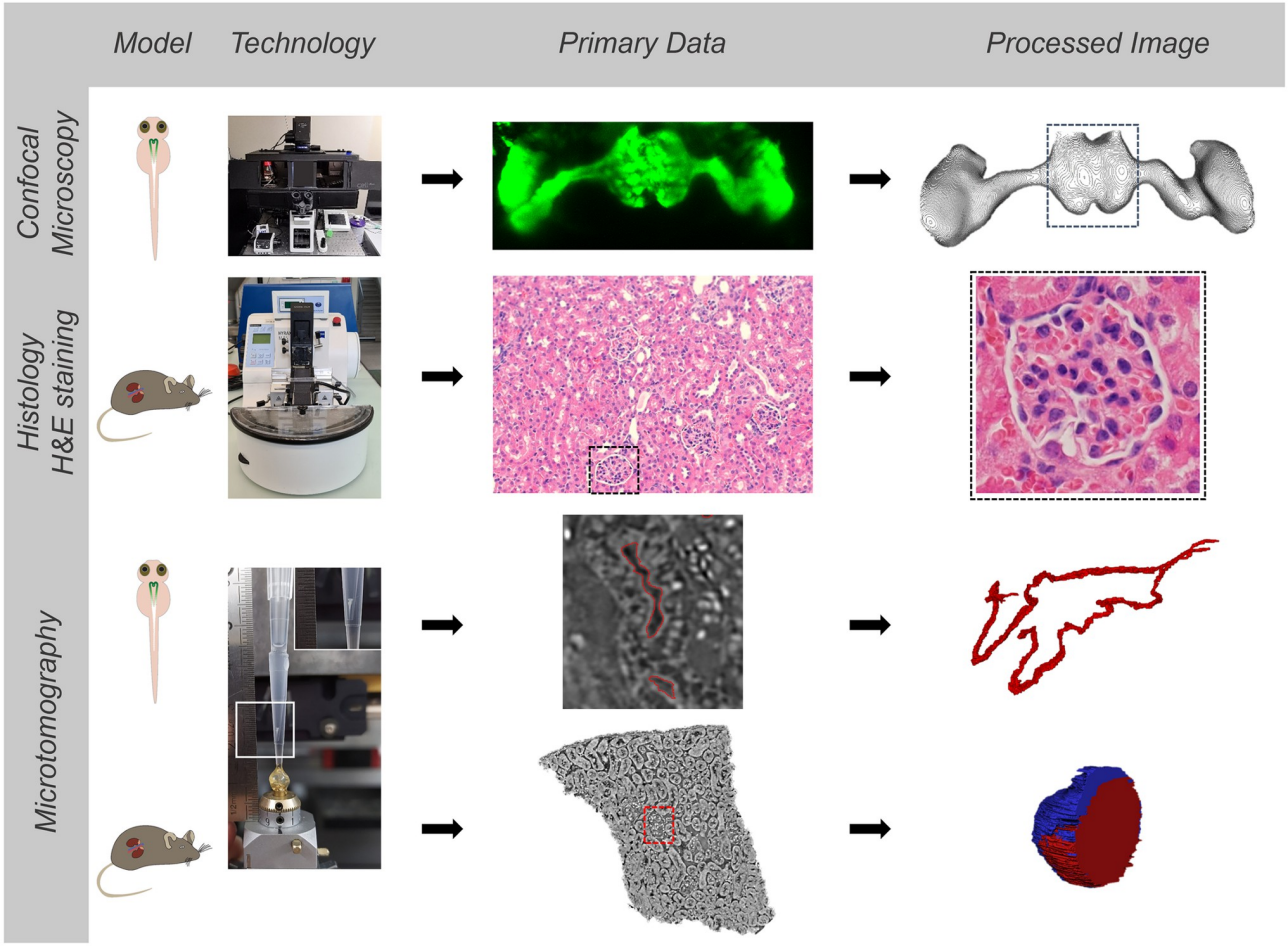
Analytical technologies, workflow, and outcome. Summary of employed imaging strategies to study toxic effects using zebrafish pronephros and mouse kidney biopsies. Methods include confocal laser scanning microscopy, synchrotron radiation-based computed tomography (SRμCT), and semiquantitative image analysis of H&E/PAS stained histology slides.

For this study, gentamicin sulphate, a well-established nephrotoxic antibiotic drug, was chosen as a model compound to induce acute toxicity in proximal tubules [20]. Several studies in ZFL have demonstrated functional impairment of glomerular filtration upon gentamicin treatment [19, 21]. These results correlate with clinical findings [22]. We, therefore, treated ZFL with nephrotoxic concentrations of gentamicin and used a transgenic zebrafish reporter line that expresses enhanced green fluorescent protein (eGFP) in the glomerulus/proximal convoluted tubule cells [23]. Glomerular impairment was visualized by confocal microscopy. SRμCT [24–27] was used for three-dimensional visualization of ZFL and mouse kidney biopsies and the semiquantitative evaluation of morphological changes. By SRμCT, a label-free visualization of anatomical structure can be achieved [24], in our case, at 0.65 μm pixel size. A scheme of workflow and methods is provided in Fig 1. To our knowledge, SRμCT was not used previously to assess renal damage. We, therefore, used drug-treated zebrafish larvae and animals to visualize and quantify structural alterations of the kidney after exposure to the nephrotoxin gentamicin.

## Materials and methods

### Animals

Five- to six-week-old C57BL/6JRj mice were purchased from Janvier Laboratories (France). The animals were housed in pathogen-free conditions at the animal facility of the Department of Biomedicine of the University Hospital Basel. All methods used on the animals were chosen to minimize pain. In addition, animals were monitored daily during gentamicin treatment using a score sheet. Zebrafish husbandry and intravenous injections were done as described [8]. Animal and zebrafish experiments were carried out in accordance with Swiss animal welfare regulations. They were approved by regulatory authorities of the Canton Basel Stadt (License 2755_29404 for mice/ 1027H, 1014HE2, 1014G for zebrafish studies).

### Materials

Gentamicin sulfate (725 g/mol) was purchased from Biowest (Nuaillé, France). Agarose, 1-phenyl-2-thiourea (PTU) and ethyl-3-aminobenzoate methanesulfonate (MS-222, tricaine) were purchased from Sigma-Aldrich (Buchs, Switzerland). The transgenic (Tg) wt1b:eGFP ZFL line expressing enhanced green fluorescent protein (eGFP) in the glomerulus and parts of the PCT were kindly provided by Prof. Dr. Schiffer from the University of Erlangen in Germany [23].

### Intravenous injections and imaging of zebrafish larvae

Regarding terminology, we follow the life stage definitions established by Kimmel et al. [28], which defines >72 hpf old zebrafish as "larvae". In brief, eggs from adult zebrafish were collected from different parents 0.5–1 hpf and kept at a temperature of 28°C in zebrafish culture media [29].

The number of larvae in a 25-mL dish did not exceed 100. The formation of pigment cells was suppressed by adding 30 μg/ml 1-phenyl-2-thiourea (PTU) to the media. 72 and 96 hpf hatched ZFL were embedded in 0.3% agar containing PTU and tricaine (0.01%). Experiments were carried out at room temperature. Randomly chosen larvae were injected with a calibrated volume of 1 nL of 14.4, 21 and 42 mM stock solutions of gentamicin sulfate into the cardinal vein (CV) above the heart. Injected sample volume was verified by calibration measurements using a reticle mounted on a Leica SAPO binocular eyepiece. For intravenous injections, a micromanipulator (Wagner Instrumentenbau, Schöffengrund, Germany), a pneumatic Pico Pump PV830 (World Precision Instruments, Sarasota, FL, US), and a Leica SAPO microscope (Leica, Wetzlar, Germany) were applied. Larvae were immobilized in agar and laid on their back for imaging. The kidney region was imaged 24 hours post-injection (hpi) using an Olympus FV3000 inverted confocal laser scanning microscope (Olympus Ltd., Tokyo Japan) equipped with a 30X UPIanSApo (numerical aperture of 1.05) objective. Confocal images were acquired using a sequential line scan, an excitation wavelength of 488 nm (argon laser) and emission wavelengths of 500 to 540 nm.

### Signal intensity quantification and post-processing of images

Confocal microscopy images were analyzed and edited using the open-source OMERO software (version 5.4.10, https://www.openmicroscopy.org/omero/) as an image-processing program. Signal intensities (SI) in distinct organs were quantified using the open-source Fiji image analysis software (version 2.1.0/1.53c, https://imagej.net/software/fiji/) as described [8]. Controls and corresponding treatment groups were analyzed during the same experiment and using the same laser and microscopy settings. A self-written FiJi script allowed for the automatic quantification of SI based on maximum intensity projections using a selected threshold. SI are presented as fold-change normalized to the mean of the PBS-treated control. Tubular dilatation was scored semi-quantitatively by a blinded observer who evaluated PAS-stained sections of nine microscopy images of three control and three treated mice. At least 20 PCT were examined at 40x magnification.

### Sample processing for microtomography experiments

ZFL were processed for microtomography experiments as described [24]. In brief, ZFL were euthanized with tricaine methanesulfonate and fixed for one hour in 4% paraformaldehyde (PFA). Fixed specimens were dehydrated using ethanol at increasing concentrations (25%, 50%, 70%, and ≥99.8%) for 15 min each under gentle agitation and stored at a temperature of 4°C. Larvae fixed in ethanol were transferred into plastic pipette tips filled with ethanol shortly before imaging and mounted on a sample holder for imaging.

Kidney tissue samples were obtained from mice treated intraperitoneally with gentamicin at 150–170 mg/kg body weight daily for ten days. The saline-treated animals were used as control animals. After 14 days, mice were euthanized by intraperitoneal injection of 150 mg/kg pentobarbital, perfused transcardially with phosphate-buffered saline (PBS), and the kidneys were isolated. For histology examination, the right kidney was cut in half, fixed in 4% ice-cold PFA for 40 hours, washed with PBS, and transferred to 70% ethanol. For microtomography measurements, the left kidney was immediately put on dry ice after extraction, followed by -80°C freezing. Needle biopsies were prepared by punching small cylindrical samples from frozen tissue using a G18 injection needle. They were immediately transferred to ice-cold 4% PFA for half an hour. Biopsies were washed three times with ethanol, stored in >99% ethanol, and then mounted on a microtomography sample holder.

### Histology of mice kidney biopsies

Kidneys were paraffinized using a tissue processor and a tissue embedding station (TPC15, Medite Medical GmbH, Burgdorf, Germany). In brief, 3 μm paraffin tissue slices were prepared using a Thermo Scientific rotary microtome (Waltham, MA, US) and mounted on thin glass slides (Menzel, Braunschweig, Germany). Histological slices were stained with hematoxylin and eosin (H&E) or periodic acid-Schiff stain (PAS) performed by a Gemini AS automated stainer (Epredia, Portsmouth, NH, US). Stained slices were imaged using a laser microscope (Olympus Ltd., Tokyo, Japan) equipped with a 40X UPIanSApo (numerical aperture of 1.05) objective. To quantify the glomerular area, samples were blinded to limit the biased interpretation of treatment and were analyzed by up to three operators. Thirty glomeruli per mouse were visually examined. The ratio between the mean glomerulus area and the mean glomerulus area occupied by cells was determined based on manual segmentation using a Wacom Intuos pen tablet (Wacom, Kazo, Japan) and Fiji software for quantification.

### Synchrotron radiation-based microtomography analysis

Synchrotron radiation-based computed tomography (SRμCT) was performed at the ANATOMIX beamline of Synchrotron SOLEIL (Saint-Aubin, France) [30]. The undulator X-ray source was set to a magnetic gap of 8.3 mm. A 10 μm Au filter was implemented to obtain a filtered white beam with a mean photon energy of around 17 keV. The detector was placed 50 mm downstream of the sample to allow for propagation-based phase contrast. Projections were recorded with an effective pixel size of 0.65 μm. The detector consisted of a 20 μm LuAG scintillator coupled via a 10× objective to a scientific CMOS camera (Hamamatsu Orca Flash 4.0 V2, 2048×2048 pixels, 6.5 μm physical pixel size) [31]. An exposure time of 50 ms was selected, providing an average detector signal of approximately 30,000 analog-digital units (ADU) in the flat field. For tomographic imaging, 4,000 projections were recorded over 180° in flyscan mode. Each height step covered a cylindrical volume with 1.3 mm diameter and 1.3 mm height and required a scan time of approximately 3.5 minutes. Depending on alignment, one or two height steps were needed to image the whole zebrafish larva.

Tomographic reconstruction was performed using the software pipeline and computational resources available at the ANATOMIX beamline, which uses the PyHST2 software (ESRF, Grenoble, France) for the filtered backprojection step. Prior to reconstruction, projections were phase-retrieved using Paganin’s filter [32] with a kernel length of 15 μm [33]. A double flat-field correction was applied to suppress ring artefacts [33].

### Three-dimensional rendering and segmentation

The lumen of proximal tubules of ZFL and glomeruli of mouse biopsies were manually segmented using Amira software (Version 6.2.0, ThermoFisher, Massachusetts, US). The segmented volume was subsequently analyzed in Matlab (release R2020a, The MathWorks Inc., Natick, US). Tubules of treated ZFL were compared by extracting their centerlines via medial axis transformation. The medial axis consists of all points which have more than one closest point on the object’s boundary. It was extracted with the Matlab function "bwskel". The tubules were aligned based on the manually identified turning point (TP) and the geodesic distance along the centerline. The geodesic distance was determined by reducing the three-dimensional centerline coordinates to one dimension via the Isomap mapping software provided by van der Maaten et al. [34]. The length, as well as spatially-resolved diameters, cross-section areas, and SI of the areas, were calculated for each centerline and summarized.

### Statistical analysis

Statistical analysis was performed with GraphPad Prism Version 8.0.2 (GraphPad Software, San Diego, CA) using one-way analysis of variance (ANOVA) followed by Dunnett’s multi-comparison tests or unpaired two-tailed t-test analysis for direct comparisons. Where appropriate, individual data points are presented as dot plots next to the group’s average and standard deviation (SD). The number of individual ZFL and mice is indicated as (n). The statistical significance of the difference against the control population was evaluated at the 0.05 and 0.001 probability level and marked by asterisks. Data are shown as a box-and-whisker plot indicating the datasets’ minimum, maximum, median, first quartile, and third quartile.

## Results

**Fig 2:**
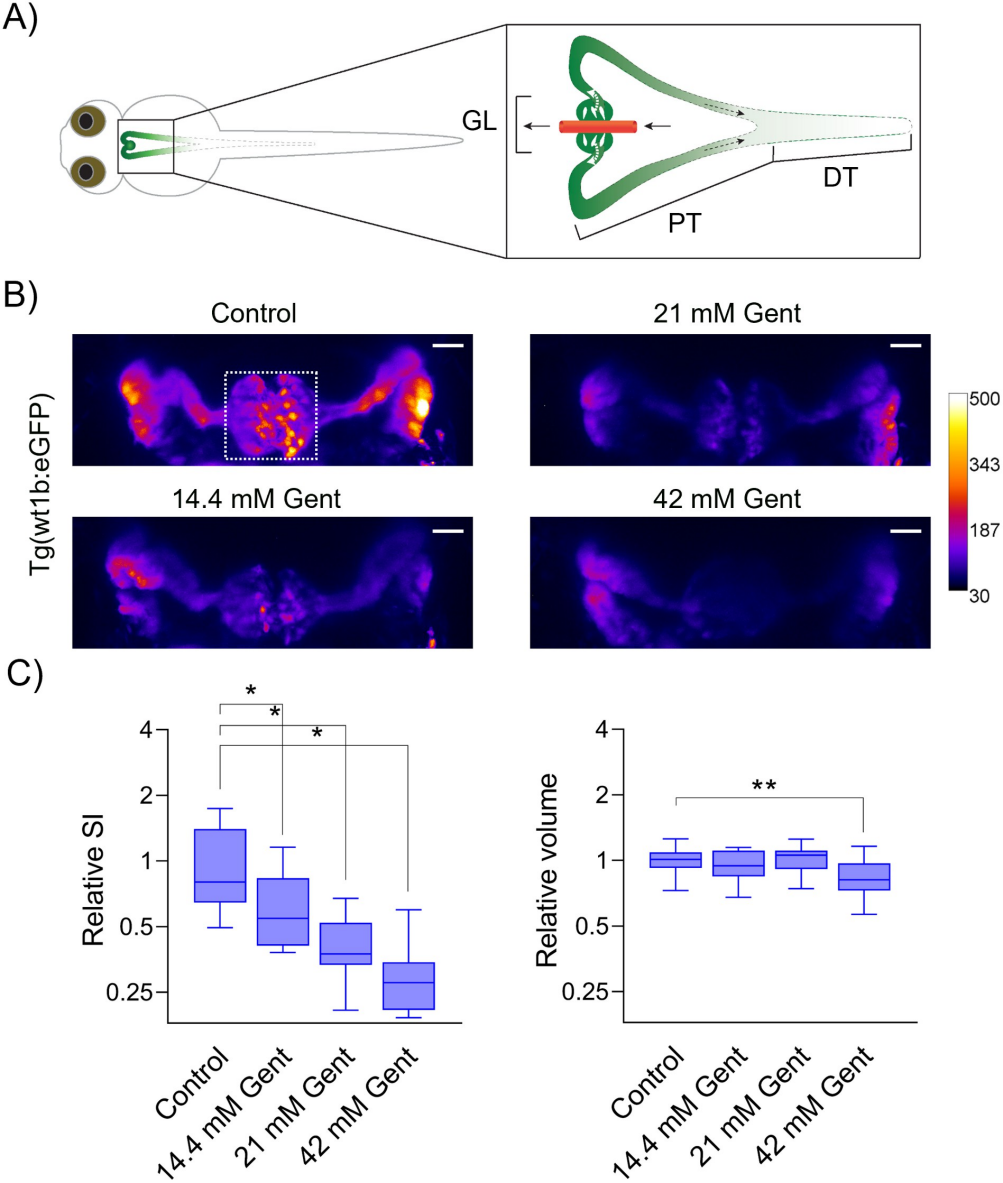
Gentamicin-induced damage to the pronephros in 96 hpf Tg(wt1b:eGFP) ZFL. (A) Schematic representation of renal pronephros in 96 hpf ZFL. GL: glomerulus, PT: proximal convoluted and straight tubule, DT: distal early and late tubule. Pronephros are fused together at the midline above the yolk and heart. Transgenic expression of eGFP leads to a fluorescent signal (green) in the glomerulus and upper parts of the tubules. (B) 72 hpf Tg(wt1b:eGFP) ZFL were injected with 1 nL of 14.4 mM to 42 mM gentamicin. Representative images of maximum projections. The color-coded heat map indicates SI, e.g. white correlates to high SI values and black to weak SI. Scale bar: 25 μm. Gent: gentamicin. (C) Relative SI and relative volume of the glomerulus (panel B, white dotted rectangle) as compared to normalized control is shown using a box-and-whisker plot, n ≥ 10 ZFL. *p < 0.001, **p < 0.05.

The zebrafish larvae’s kidney formation is illustrated in Fig 2A. The pronephros consist of two renal tubules that are glomerular fused ventral to the dorsal aorta at the larvae’s midline [16]. The pronephros can be divided into three functional units (Fig 2A). First, the blood is filtered within the glomerulus (GL). The filtrate is collected within the proximal tubules (PT) and drained through the distal tubules (DT) to the cloaca. The anatomy and morphology of the upper renal system of the ZFL can be visualized using transgenic lines expressing eGFP in the glomerulus and parts of the PT, e.g. Tg(wt1b:eGFP). Confocal laser scanning microscopy allows for the three-dimensional imaging of the glomerulus, marked in Fig 2B by a white dotted quadrant. A confocal laser scanning microscope was used for dorsal imaging. This allows for the quantitative determination of SI and three-dimensional volume calculations of the glomerulus. Results show a steady monotonic decrease in SI of eGFP in the transgenic lines as the concentrations of the injected gentamicin are increased (Fig 2C). The intensity decrease is statistically significant at the 0.001 level for the tested gentamicin concentrations. The volume of the glomerulus, calculated based on thresholding the eGFP signal, is not significantly changed for injected concentrations up to 1 nL of 21 mM gentamicin. However, for the highest injected concentration (1 nL of 42 mM), we observed a statistically significant decrease at the 0.05 level of the glomerulus volume.

**Fig 3:**
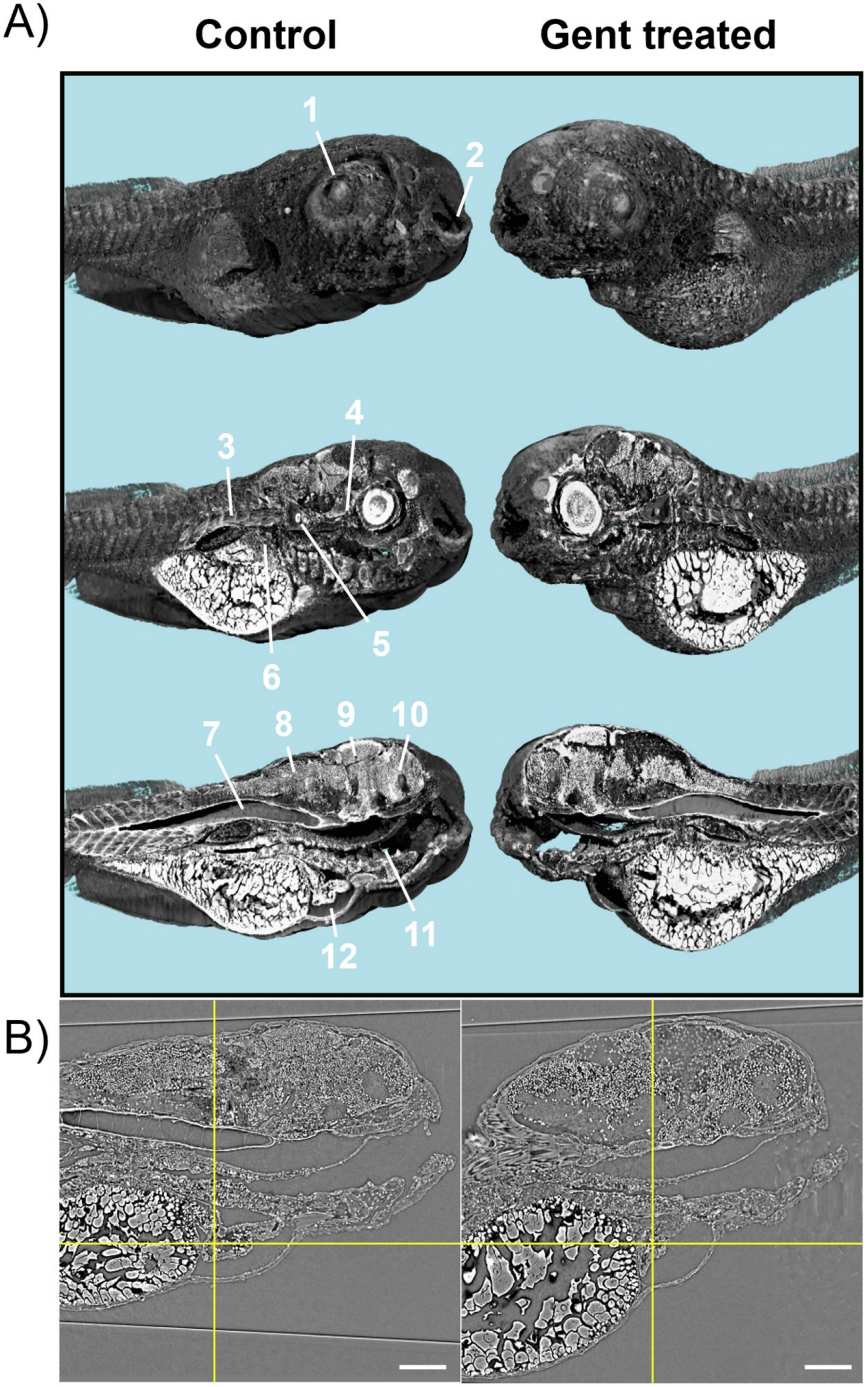
Synchrotron radiation-based computed tomography (SRμCT) of control and gentamicin-treated ZFL. (A) 3D renderings of 96 hpf ZFL at 24 hpi of 1 nL of control buffer (left panels) or 1 nL of 42 mM gentamicin (right panels) with sagittal cuts. Pixel size: 0.65 μm. Anatomical structures: 1 = eye lens, 2 = mouth, 3 = muscle tissues, 4 = optical nerve, 5 = otoliths, 6 = liver, 7 = notochord, 8 = hindbrain, 9 = midbrain, 10 = frontbrain, 11 = intestine, 12 = heart. (B) Representative virtual slices through the heart chambers (yellow reticle) of control (left) and 1 nL of 42 mM gentamicin (right) treated ZFL. Scale bar: 100 μm.

In Tg(wt1b:eGFP) ZFL, eGFP expression is limited to the glomerulus and the proximal tubules. To analyze the tubular system more completely, we used SRμCT with voxels of (0.65 μm)^3^ (Fig 3). As observed, fixation of the samples with PFA was necessary to avoid the formation of gas bubbles in aqueous buffers upon X-ray irradiation. Fig 3A shows two stacked tomograms of control and treated ZFL (1 nL of 42 mM gentamicin injected, 24 h incubation). Applying Paganin’s filter, body surfaces and inner organs can be visually inspected after 3D surface rendering. Gentamicin-treated ZFL shows malformations, i.e. a pronounced body curvature. A prominent feature is the enlarged pericardial sac, indicative of cardiac edema caused by acute kidney injury (Fig 3B).

**Fig 4:**
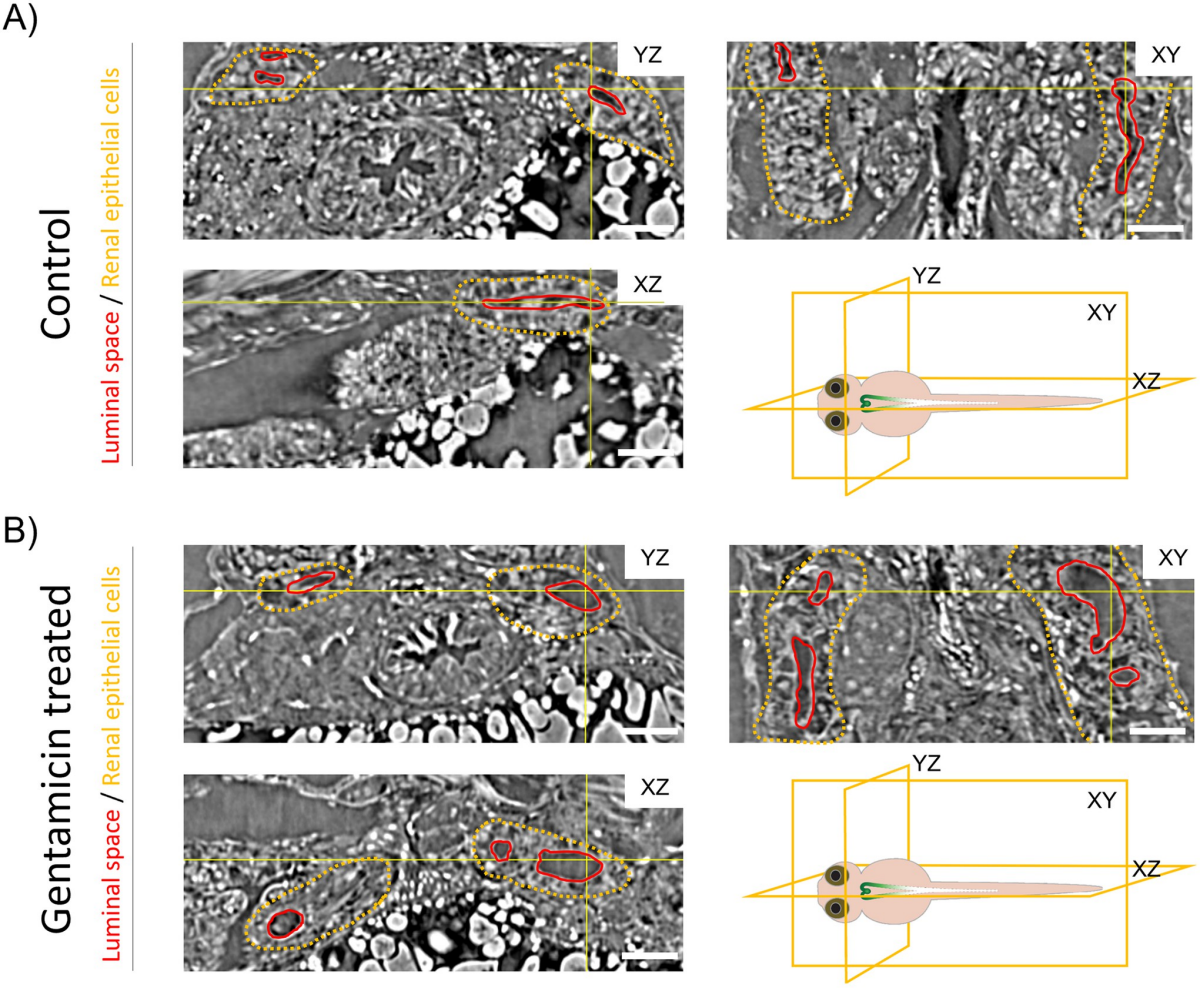
Microtomography-based visualization of the ZFL pronephros. **(A)** Orthogonal sections from the 4 dpf ZFL through the microtomography volume of PBS (control) and treated with 1 nL of 42 mM gentamicin (24 hour incubation). Yellow lines show the position of the virtual slices, which intersect at the reticle point located in the luminal space. Luminal space is outlined in red, renal epithelial cells are in yellow. Schematic illustrations indicate the cutting planes. Scale bar: 100 μm.

Virtual slices of the processed data through sagittal (XZ), traverse (YZ) and coronal (XY) planes allow for the manual localization of the renal tubules. Representative images are shown in Fig 4. The latter has a weak SI and is easily identifiable. Renal epithelial cells have diverse SI, such as cell nuclei (white), cell membranes (dark grey) and cytosols (black). Comparing renal tubules of the gentamicin treated with control ZFL, a clear enlargement of the tubular lumina in the PT region was observed.

**Fig 5:**
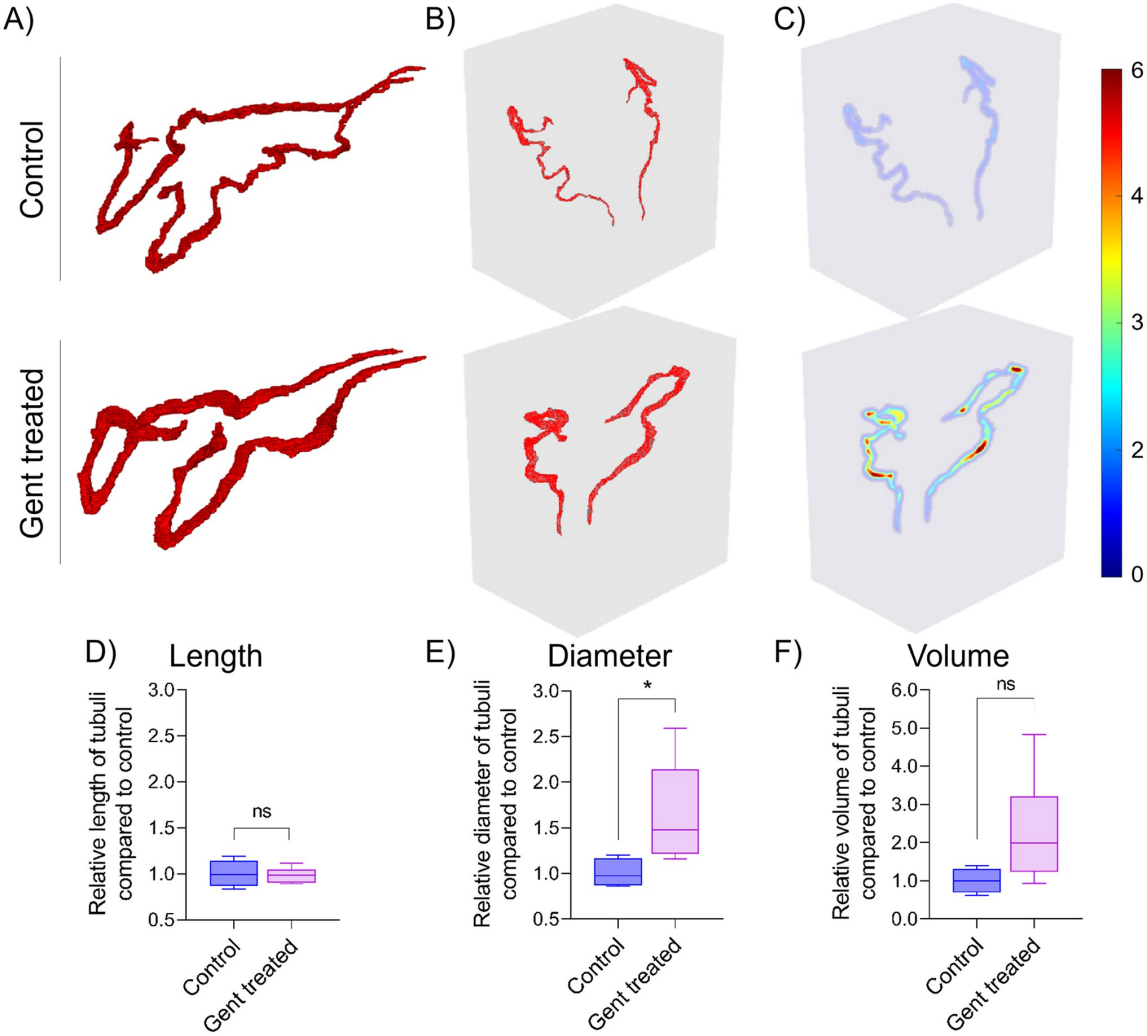
SRμCT-based reconstruction and quantification of ZFL proximal tubules. Rendered proximal renal tubules of control and gentamicin-treated ZFL (1 nL of 42 mM gentamicin, analysis 24 hpi) are shown in (A) lateral and (B) ventral projection. (C) Color-coded heat map, with values at the centerline corresponding to the diameter of the luminal area (blue: 0 μm; dark red: 6 μm) of tubules shown in B. (D, E, F) Semiquantitative analysis of length, diameter, and volume of treated ZFL tubules (n = 8) in terms of fold change as compared to the mean of control ZFL tubules (n = 4). Box plots are shown. *p < 0.05. ns: p > 0.05.

To quantify aberrations in the morphology of proximal tubules of gentamicin-treated ZFL, renal tubules were manually segmented in two dimensions (XY and YZ), and diameters along the centerline were determined. A representative 3D rendering of control and treated renal tubules is shown in Fig 5. Confirming observations from the virtual slices in Fig 4, the shape and thickness of the volume of control and treated tubules differ in both a lateral (Fig 5A) and ventral (Fig 5B) view. Based on a three-dimensional segmentation and evaluation, the thickness of renal lumina (Fig 5C) can be represented using a color-coded map. Red indicates enlarged diameters of the luminal space (6 μm), which was observed in gentamicin-treated animals only. Analysis of eight treated and four control ZFL showed no difference in the length of the segmented tubules (Fig 5D) but a statistically significant 1.75-fold increase in diameter (Fig 5E and S1 Fig). Differences in the entire volume are not statistically significant (Fig 5F).

**Fig 6:**
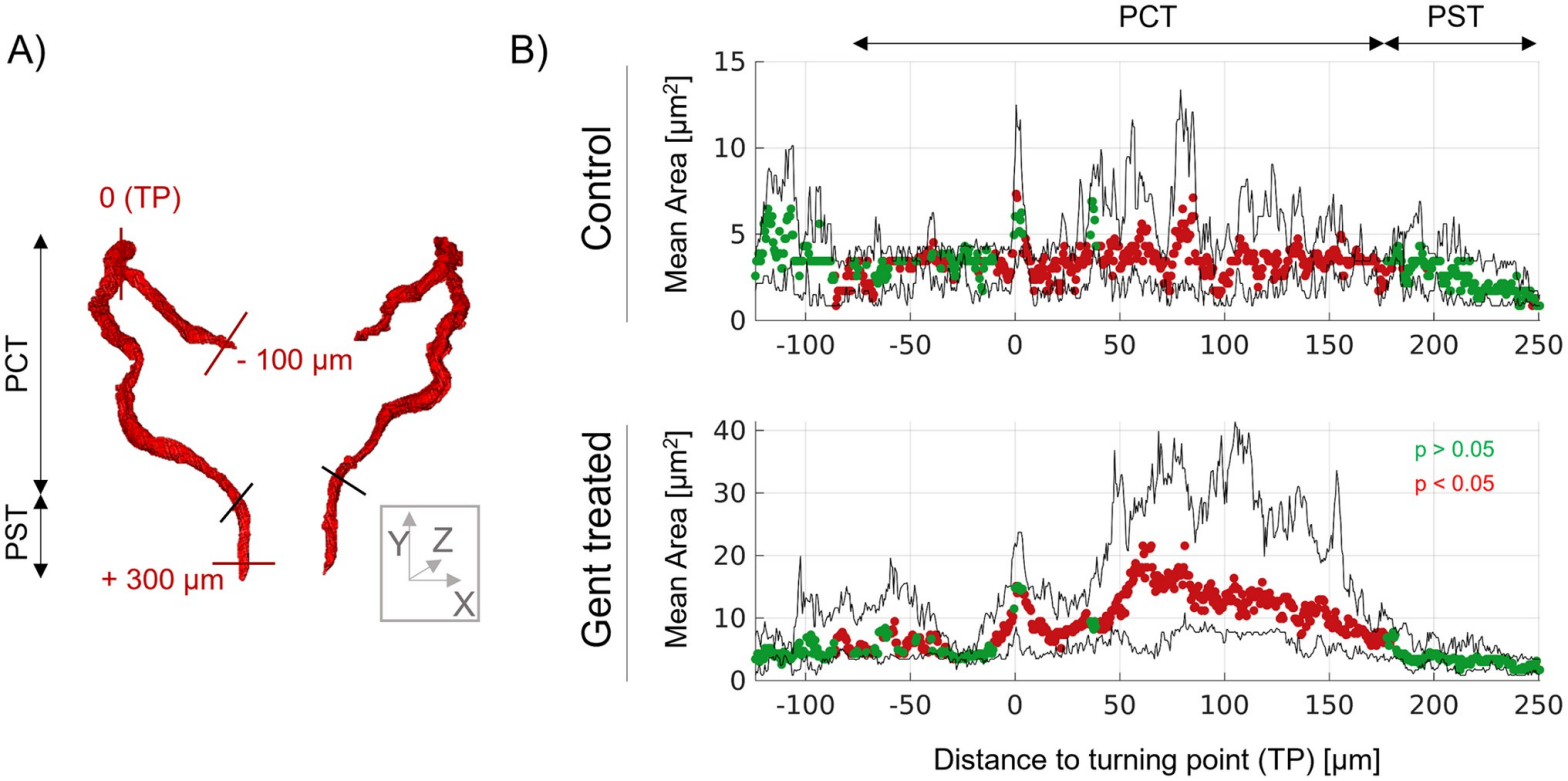
Localization of gentamicin-induced tubular damage. (A) Rendered renal tubules are divided into PCT and PST. Distances to the turning point (TP, defined as zero) are indicated in the direction of the glomerulus (negative values) and towards the cloaca (positive values). (B) The plot of tubule cross-section area against distance from TP. Gentamicin treatment: 1 nL of 42 mM gentamicin, analysis 24 hpi injection. Symbols: mean cross-sectional area, color coded with respect to significant differences between control (n = 4) and gentamicin (n = 8) treated ZFL. Green symbols: p > 0.05. Red symbols: p < 0.05.

To quantify the extent of tubular damage in distinct tubule sections, the segmented proximal tubule was manually divided into PCT and proximal straight tubules (PST), as shown in Fig 6A. Furthermore, the centerline along the tubules was projected onto a coordinate system (Fig 6B). The turning point (TP) was defined as zero. The distance between TP and the glomerulus (neck) is approximately 100 μm essentially in the *x*-direction, whereas the last segmentable lumen of the nephros is located around 300 μm with an overall orientation in the *y*-direction. The area along the center line was plotted for control and gentamicin-treated ZFL. Semiquantitative analysis of distinct regions (–75 μm to 175 μm) reveals a significant increase in the tubular lumen of gentamicin ZFL in the PCT but not in the PST.

**Fig 7:**
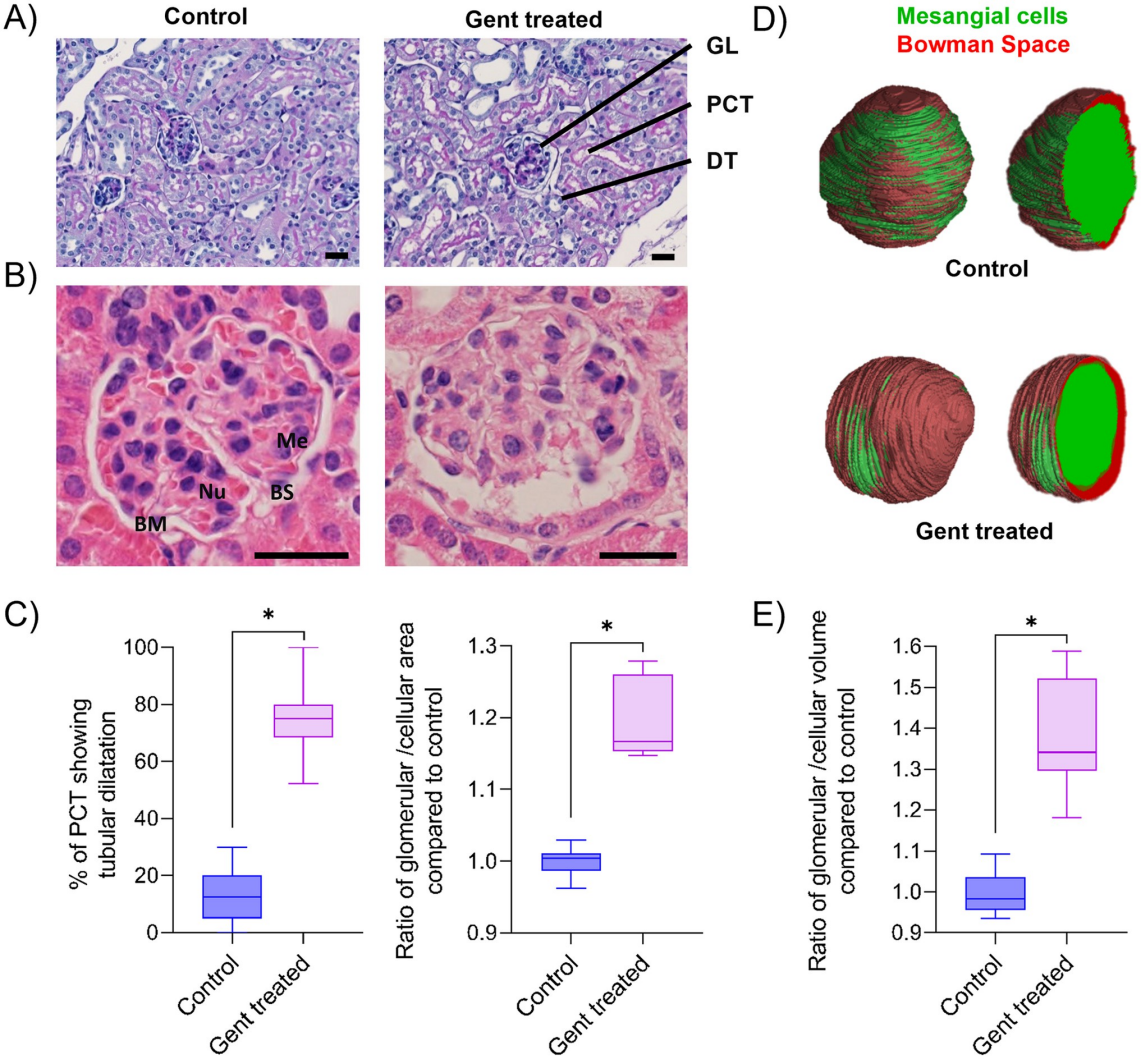
2D and 3D assessment of kidney damage in gentamicin-treated mice. (A) Representative PAS-stained histology slices (3 μm) from a kidney biopsy of a control and a gentamicin-treated mouse. Gentamicin treatment: daily i.p. injections for ten days of 150 mg gentamicin. Glomerulus = GL, proximal convoluted tubules = PCT, distal tubules = DT. (B) Representative images of an H&E stained 3 μm histological slice of a glomerulus of control and a gentamicin-treated mouse. Mesangial cells = Me, Nuclei = Nu, basal membrane = BM, basal space = BS. (C) Semiquantitative analysis of % of PCT showing tubular dilatation and the ratio of glomerular to cellular area normalized to the mean of the control. 180 PCT of three mice (control or treated) and 30 glomeruli of six mice (control or treated) were analyzed, respectively. (D) SRμCT analysis and visualization of a representative 3D rendered glomerulus of a control and a gentamicin-treated mouse. Mesangial cells are labelled in green, and Bowman’s space in red. (E) SRμCT-based analysis of the ratio of glomerular to cellular volume as compared to the mean of the control. Box plots are shown of five manually segmented glomeruli of three mice each. *p < 0.01. Scale bar: 25 μm.

In a next step, extracted mouse kidneys were subjected to histology and SRμCT analyses (Fig 7). In contrast to ZFL, the mouse kidney cortex contains a multitude of nephrons (i.e. glomeruli and renal tubules). Histological examination of periodic acid-Schiff (PAS) stained 3 μm-thin tissue sections reveals distinct differences between control and treated animals. Compared to the control, gentamicin-treated kidney biopsies show enlarged PCT lumina, whereas DT are unaffected. More detailed studies focusing on glomeruli are depicted in Fig 7B. Hematoxylin and eosin (H&E) stained kidney sections reveal a homogenous arrangement of mesangial cells (Me) and their Nuclei (Nu) in control animals. The basal membrane (BM) is visible and present, and the Bowman’s space (BS) is narrow and uncolored.

In comparison, kidney sections from gentamicin-treated mice show a heterogeneous pattern (Fig 7C right panel). It has an enlarged BS, and the BM is hardly present. Further, cellular waste and debris are located within the glomerular space.

For semiquantitative analysis, histology images of 30 glomeruli of three mice from each treatment group were compared (Fig 7B) and further evaluated. 75% of analyzed PCT of gentamicin-treated mice showed tubular dilatation compared to 13% of the control group (Fig 7C, left panel). The ratio of the glomerulus area divided by the cellular area of the same glomerulus shows a significant 1.2-fold increase (p<0.01) in the gentamicin group (Fig 7C, right panel). The mean glomerular area of the control group was (3 337 ± 322) μm^2^, and the corresponding area of the gentamicin group was (3 524 ± 295) μm^2^ (ns). The mean cellular area of the control group was (2 866 ± 321) μm^2^ vs (2 565 ± 226) μm^2^ (ns) of the gentamicin group. The morphological changes observed using SRμCT to obtain 3D representations of glomeruli are similar to 2D analysis, but the volumes are statistically significant (p<0.01). Two representative, segmented glomeruli are shown colored in red (Bowman’s space) and green (mesangial cells of the glomerulus). Next, the quantitative analysis of five glomeruli from three control and three treated mice was performed (Fig 7E). Gentamicin leads to a 1.4-fold increase in the ratio of the glomerular to cellular volume. Thus, using this alternative imaging method, we could demonstrate that the glomerular volume is significantly (p < 0.01) elevated in the gentamicin-treated group (81 083 μm^3^ ± 19 479 μm^3^ vs 25 379 μm^3^ ± 7 756 μm^3^). In contrast, the cellular volume is not significantly (p = 0.09) reduced compared to the control group (121 265 μm^3^ ± 15 682 μm^3^ vs 131 994 μm^3^ ± 22 455 μm^3^).

## Discussion

Xenobiotics such as the aminoglycoside antibiotic gentamicin accumulate within renal tissue during their elimination and cause nephrotoxicity. We used in the present study ZFL as a vertebrate model to assess the impact of gentamicin on kidney morphology. Findings were compared to results from SRμCT of kidney biopsies of gentamicin-treated mice. We could demonstrate that extrapolation of findings in ZFL to rodents is possible thus confirming similarity of anatomical and functional structures of the developing pronephros of ZFL and corresponding structures in mammals, including humans [16, 35].

Zebrafish larvae offer the possibility to inject a defined dose into the bloodstream using an appropriate injection platform. Hereby, an initial concentration is known, and blood concentration follows a kinetic profile as done with other in vivo (vertebrate) studies. Bathing zebrafish larvae in a toxin solution is an easier approach but lacks information about initial blood concentrations. Therefore, we believe that i.v. injection of nephrotoxins into zebrafish larvae is the better and more vertebrate-relevant approach for nephrotoxicity studies. This approach was also confirmed in other literature reports using zebrafish larvae in nephrology [12, 36].

The concentration used for zebrafish studies in this study was chosen based on clinical relevant plasma concentrations in the range of 2 to 20 μM gentamicin, daily doses for seven to ten days [37, 38]. For serious bacterial infections, a longer course of therapy might be necessary. With respect to zebrafish, the model drug was intravenously injected into the cardinal vein using an injection volume of 1 nL, corresponding to approximately 0.8% of the total blood volume of a 96 hpf ZFL [8]. This was a prerequisite to obtain defined exposure of ZFL towards gentamicin and to avoid potential interference with intestinal metabolism. Trough to peak plasma concentrations reached in our study (single bolus injection) are therefore estimated to cover a range of 12 to 350 μM gentamicin.

Using a transgenic zebrafish line expressing eGFP in the glomerulus and parts of proximal renal tubules under the wt1b promoter [23], mean SI in the glomerulus was assessed 24 hours post gentamicin injection. A concentration-dependent SI decrease indicated a cytotoxic effect interfering with eGFP expression. These findings are in agreement with previous studies using wild-type ZFL at three dpf [21]. Functional assays using FITC-DX 70 kDa showed that gentamicin-induced nephrotoxicity lead to impaired glomerular filtration resulting in an acute kidney injury with loss of cell polarity of proximal tubules [12, 18]. Exposure towards different types of nephrotoxins led to impaired kidney function (e.g., reduced clearance of FITC-dextran) and upregulation of kidney injury marker genes [21]. It should be noted that a decrease in the fluorescent SI of a marker protein is not necessarily indicative of renal damage. We therefore used synchrotron radiation-based microtomography with a pixel size of 0.65 μm to visualize morphological aberrations of renal tubules. It was thus possible to study unstained organs, including renal tubules and the changes in the morphology of the heart. In contrast to previous studies using a mean photon energy of 10.5 keV [24], the central photon energy used in this study was higher, at around 17 keV. However, this did not offer additional advantages as images were of similar quality. It is tempting to speculate that recent laboratory-based tomography instruments providing phase-contrast capabilities, liquid metal X-ray source technology, and X-ray detector optics may provide a spatial and density resolution comparable to the present study [25]. This will provide opportunities for a broader applicability of the presented tomography-based approach, although at the expense of longer acquisition times.

The toxic effect of gentamicin caused impairment of larval development in general, leading to body curvature and cardiac edema [18], in addition to renal impairment. This finding is similar to previous studies using the same transgenic line to assess drug-induced kidney malformation and other toxicants [39, 40]. The pronephric kidney of the ZFL is located caudal of the head and above the yolk. The glomerulus is located above the heart. The microtomography cross sections easily reveal the pronephros since it is a pair-wise organ with a low x-ray absorbing fluid-filled luminal space surrounded by a high x-ray absorbing cell layer.

In contrast, the segmentation of PCT tubules with a diameter of two to 30 pixels from the surrounding tissue was a challenge. We had to rely on manual segmentation, since automated procedures using dedicated software tools were not applicable. Consequently, we focused on determining volumetric parameters (e.g., tubule diameter, length, area, and volume). This approach allows for a label-free microtomography imaging of organ-specific morphological aberrations caused by nephrotoxin in ZFL. Thus, in contrast to previous studies, no density-based labelling strategies using density-rich elements such as zinc, iron and copper [41] were employed.

This study used SRμCT analysis to visualize and analyze the pronephros of ZFL. Glomeruli were not segmented, since mesangial cells are densely packed within Bowman’s capsule. Therefore, no separation of cell layers was possible. In mice, renal tubules were analyzed using 2D tissue sections. This was sufficient to allow for rapid and precise evaluation of tubular toxicity.

There is a strong correlation between findings in the ZFL and rodents. In agreement with ZFL findings, histology sections of H&A/PAS-stained mouse renal biopsies revealed gentamicin-induced damage of glomeruli and an increase of the lumen of proximal tubules. This correlated with findings in the rat [42–44] and was corroborated by three-dimensional reconstruction of murine glomeruli based on SRμCT analysis. It is noteworthy that 3D image reconstruction allowed for a visualization of the whole glomerulus, which is superior to the analysis of histology sections due to the asymmetric distribution of collapsed apoptotic cells and capillaries within the damaged glomerulus [45]. Functional studies in gentamicin-treated mice confirms renal impairment by elevated kidney function biomarkers, such as serum creatinine, blood urea nitrogen and urea together with increased cytokine levels, such as interleukin-6 and tumor necrosis factor-alpha (TNF-α) [46, 47].

## Conclusion and outlook

ZFL is a useful *in vivo* vertebrate screening model that is frequently used in pharmacological, toxicological and nanomedicine research [9, 21]. The study using gentamicin as a model nephrotoxin reveals a similarity between morphological aberrations in ZFL and rodent kidney. Glomerular and proximal tubular damage can be visualized by confocal microscopy using the Tg(wt1b:eGFP) fish line that expresses GFP in the glomerulus and parts of the PCT. Alternatively, label-free SRμCT-based tissue analysis can be employed. We, therefore, propose ZFL as a translational model to assist in the extrapolation from cell-culture-based test systems to mammals. The approach is cost-effective, in agreement with 3R principles of animal welfare, and could therefore be of interest for identifying potential nephrotoxins in drug discovery.

## Supporting information

S1 Fig 3D reconstruction and quantification of four control and eight gentamicin-treated ZFL pronephros. (DOCX)

S1 Graphical abstract (TIF)

S1 Text (TXT)

## Abbreviations

Dpf: days post-fertilization
eGFP: enhanced green fluorescent protein
FITC: Fluorescein isothiocyanate
H&E: hematoxylin and eosin
hpf: hours post-fertilization
hpi: hours post-injection
PAS: periodic acid-Schiff stain
PCT: proximal convoluted tubule
PST: proximal straight tubule
PTC: proximal tubule cell
PTU: 1-phenyl-2-thiourea
SI: Signal intensity
SRμCT: synchrotron radiation-based computed tomography
Tg: transgenic
ZFL: zebrafish larva(e)
μCT: micro computed tomography
