# Supplementary material for: Zebrafish (Danio rerio) larvae as a predictive model to study gentamicin-induced structural alterations of the kidney
Source: PLoS One. 2023 Apr 20;18(4):e0284562. doi: 10.1371/journal.pone.0284562 (PMC10118166; doi:10.1371/journal.pone.0284562)
Supplement: S1 Fig — (DOCX) [file pone.0284562.s001.docx]

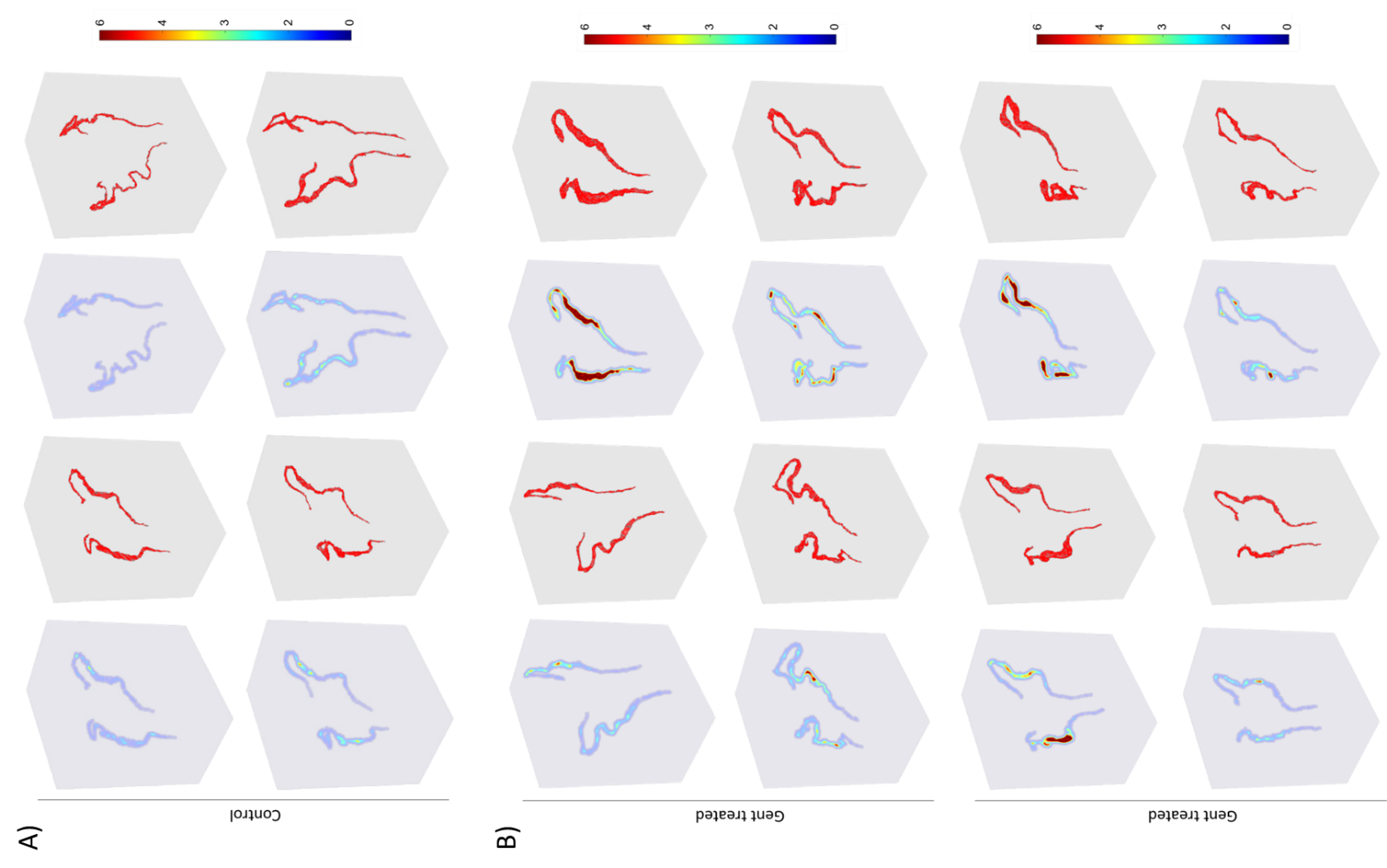


***SFig 1: 3D reconstruction and quantification of four control and eight gentamicin-treated ZFL pronephros.*** *(A) The colour-coding heat map indicates the tubules' diameter of the luminal area (blue: 0 µm; dark red: 6 µm). Rendered renal tubules of the control group are shown in red colour. (B) Same visualization as in (A), but renal tubules of gentamicin treated ZFL are shown.*
