# Supplementary figures and images for: Zebrafish (Danio rerio) larvae as a predictive model to study gentamicin-induced structural alterations of the kidney
Source: PLoS One. 2023 Apr 20;18(4):e0284562. doi: 10.1371/journal.pone.0284562 (PMC10118166; doi:10.1371/journal.pone.0284562)

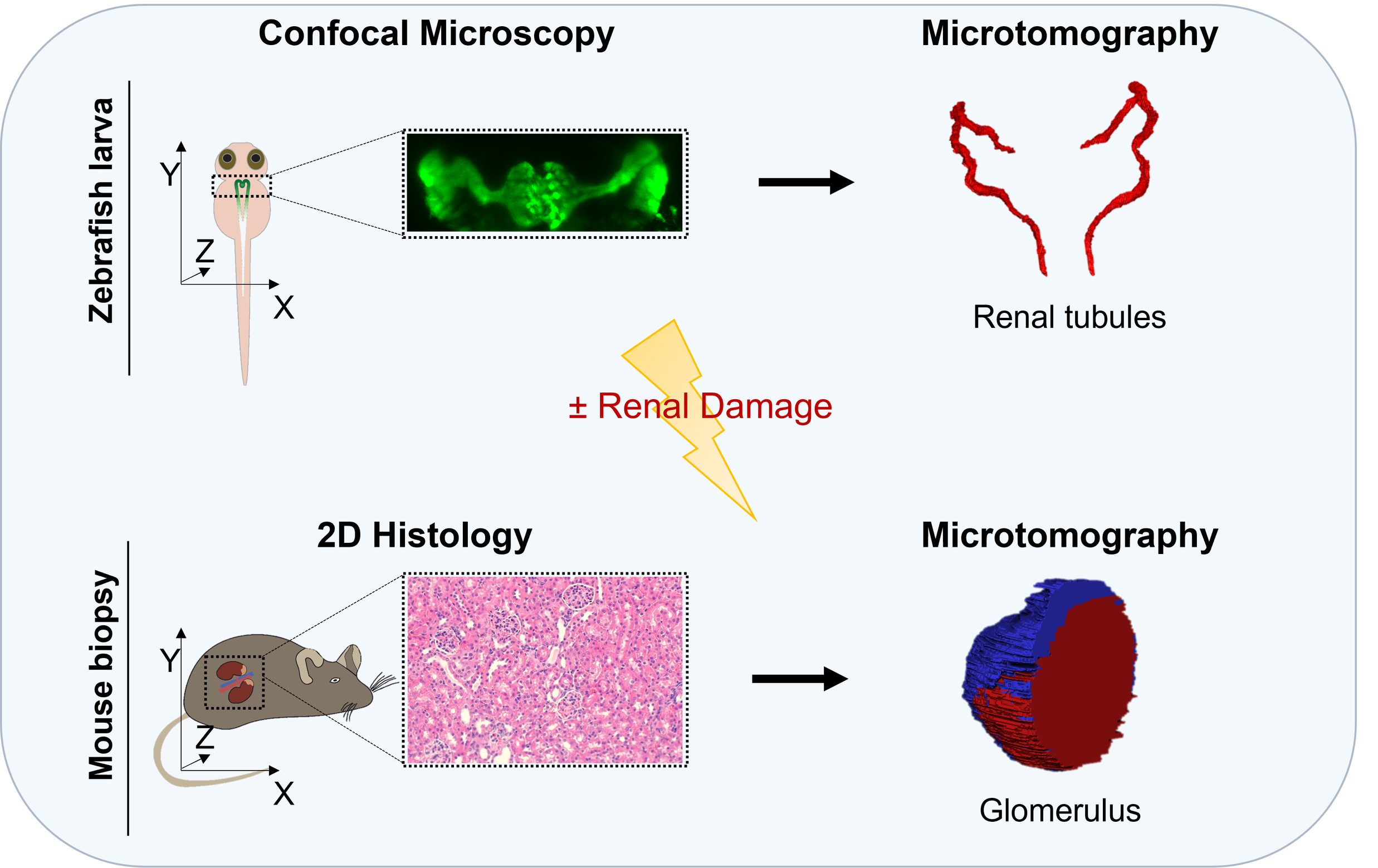

Supplement: S1 Graphical abstract — (TIF) [file pone.0284562.s002.tif]
